# Remarkable Inhibition Efficacy of a Compound Plant Essential Oil Disinfectant Against Bacteria, Viruses, and Mycoplasmas

**DOI:** 10.3390/vetsci12100978

**Published:** 2025-10-11

**Authors:** Ming Guan, Tao-Ni Zhang, Cheng Lu, Jin-Xin Zhou, Ri-Wang Yang, Xuan-Ming Dong, Cheng-Yu Zhang, Qi Wang, Wen-Qing Zhao, Yu Zhang, Tian-Chao Wei, Jian-Ni Huang, Teng Huang, Mei-Lan Mo

**Affiliations:** 1 College of Animal Science and Technology, Guangxi University, Nanning 530004, China; 2 Guangxi Zhuang Autonomous Region Engineering Research Center of Veterinary Biologics, Nanning 530004, China; 3 Guangxi Key Laboratory of Animal Breeding, Disease Control and Prevention, Nanning 530004, China

**Keywords:** plant essential oil, disinfectant, bacteria, virus, mycoplasma, antimicrobial

## Abstract

Infectious diseases caused by pathogenic microorganisms have caused serious economic losses to animal husbandry, and the use of appropriate disinfectants is crucial for eliminating these pathogens. Plant essential oils (PEOs), as natural bioproducts, have the characteristics of safety, non-toxicity, and broad spectrum. In this study, the inhibition efficacies against bacteria, viruses, and mycoplasmas of a compound PEO disinfectant (designated as Lei-Huo-Fu) were evaluated through determination of minimum inhibitory concentration (MIC) and bactericidal rate against *Escherichia coli*, *Staphylococcus aureus*, and *Salmonella* spp.; inactivation rate of avian infectious bronchitis virus (IBV); as well as determination of MIC of *Mycoplasma gallisepticum* (MG) and *Mycoplasma synoviae* (MS). The results showed that the MIC values of the PEO disinfectant against *Escherichia coli*, *Staphylococcus*, and *Salmonella* spp. were as low as 0.00375 µg/mL to 0.03 µg/mL. The bactericidal rates against *Escherichia coli*, *Staphylococcus aureus*, and *Salmonella* spp. reached over 95% within 30 min at a concentration of 0.03 µg/mL. For three dominant prevalent genotype strains of LX4-type, Mass-type, and Taiwan-type of IBV, the inactivation rates achieved by the PEO disinfectant at a concentration of 0.015 µg/mL and a disinfection time of 30 min were all above 99.9%. The MIC of the PEO disinfectant against MG and MS was 0.001875 µg/mL and 0.00375 µg/mL, respectively. In conclusion, the compound PEO disinfectant (Lei-Huo-Fu) has significant inhibitory effects on bacteria, viruses, and mycoplasmas, and possesses broad-spectrum antimicrobial activity. However, it is important to note that these findings are based on laboratory assays, and the efficacy in practical settings, along with the exact mechanisms of action, require further investigation. In this study, the compound PEO disinfectant demonstrates promising in vitro efficacy, suggesting its potential as a candidate for development into a safe, efficient, and natural disinfectant, pending further validation.

## Simple Summary

To address the growing challenge of antimicrobial resistance, this study developed a novel synergistic disinfectant, “Lei-Huo-Fu,” by blending Litsea cubeba, cinnamon, and star anise essential oils. This compound overcomes inherent limitations of single-component plant essential oils—such as high volatility, poor solubility, and a narrow spectrum—by delivering potent triple broad-spectrum efficacy. It demonstrates exceptional activity against bacteria (*Escherichia coli*, *Staphylococcus aureus*, and *Salmonella* spp.), with MIC values as low as 0.00375–0.03 µg/mL and over 95% bactericidal rates within 30 min. Furthermore, it effectively inactivates major IBV coronavirus strains at a rate of 99.97–99.99% and uniquely targets cell wall-deficient mycoplasmas (MG/MS) with MICs of 0.001875–0.00375 µg/mL. This safe, efficient, and natural disinfectant shows significant potential for controlling zoonotic pathogens and improving animal husbandry environments.

## 1. Introduction

With the scale and intensification of the breeding industry, the incidence of animal infectious diseases caused by pathogenic microorganisms is also on the rise, causing huge economic losses to the global animal husbandry industry [1]. In addition, some pathogenic microorganisms (such as *Escherichia coli*, coronavirus, etc.) are also pathogens of zoonoses, posing a serious threat to food safety and human health [2]. Antibiotics have long been the primary means of controlling the diseases. However, the overuse of antibiotics has led to the development of antibiotic-resistant pathogens [3]. At present, the problem of drug resistance of pathogenic microorganisms is becoming increasingly serious globally. What is more serious is that the drug resistance of pathogenic microorganisms is transmitted to humans through the food chain [4]. Recognizing the various negative effects of extensive antibiotic use—such as gut microbiota imbalance, drug residues, and increased resistance—the European Union banned the addition of growth-promoting antibiotics in animal feed in 2006, and China made the same ban in 2020 [5,6,7,8,9]. The source of infection, the route of transmission, and susceptible animals are the three basic factors in the epidemic of infectious diseases. The use of appropriate disinfectants can effectively eliminate pathogens, cut off transmission routes, and improve the farming environment, thus preventing and controlling disease outbreaks [10]. Although traditional chemical disinfectants have remarkable bactericidal effects, they generally have problems such as strong irritation, strong corrosiveness, easy development of drug resistance, environmental pollution, etc. [11]. Therefore, the exploration and development of efficient, safe, low-toxicity, and broad-spectrum disinfectants is of vital importance for creating a favorable living environment for animals, and is of great significance to both animal health as well as public health [12]. Therefore, there is an urgent need to develop green and safe alternatives.

Plant essential oils (PEOs), which are volatile aromatic compounds extracted from various parts of plants, possess characteristics such as natural origin, safety, diverse bioactivity, environmental friendliness, and pleasant fragrance [13,14]. These properties offer significant advantages over traditional disinfectants. The compound PEO disinfectant investigated in this study can be classified as a non-thermal, non-oxidizing chemical agent. Unlike disinfectants that rely on thermal energy (e.g., pasteurization) or strong oxidative properties (e.g., chlorine, peroxides), its efficacy is derived from the inherent biological activity of its natural components, positioning it as a promising green alternative. It is understood that PEOs is commonly used to prevent or treat diseases [15]. Extensive research has documented the broad-spectrum antimicrobial potential of PEOs across diverse pathogen categories. For instance, it has been reported that Cinnamomum cassia essential oil (CEO) and Litsea cubeba essential oil (LEO) have inhibitory effects against *Salmonella* (*S. pullorum*), and the combination of essential oils can disrupt the structural integrity of Salmonella, inhibit biofilm formation, and reduce extracellular polysaccharide content and bacterial aggregation ability, thereby achieving inhibitory effects on *Salmonella* [16]. Similarly, PEOs has shown significant antifungal efficacy. The essential oils of thyme, mint, and lavender showed inhibitory effects against *Microsporum canis* and *Trichophyton mentagrophytes* isolated from cats and dogs with clinically infected dermatophytosis [17]. It has been demonstrated that thymus vulgaris essential oil (TEO) was found to inhibit feline coronavirus (FCoV) replication at 27 µg/mL [18]. Furthermore, studies have shown that essential oils such as Origanum vulgare, Satureja hortensis, and Thymus vulgaris have significant ovicidal activity against gastrointestinal nematodes in sheep [19]. Notably, compared to Tylvalosin alone, the combination of Tylvalosin and Eucalyptus oil exhibited enhanced therapeutic efficacy in the treatment of Mycoplasmosis in chickens, significantly reducing the severity of chronic respiratory disease (CRD) caused by *M. gallisepticum* in broiler chickens [20]. However, so far, few studies on PEO disinfectants have been reported. Therefore, it is extremely necessary and urgent to develop PEO disinfectant.

Single essential oils face challenges like strong volatility, poor solubility, and a narrow spectrum [21]. Critically, a significant research gap exists. While existing studies focus on single oils against specific microorganisms, systematic research on formulated disinfectants against complex pathogens (bacteria, viruses, and mycoplasma) is lacking. Research on mycoplasma inhibition is particularly weak due to their lack of a cell wall [22]. Therefore, developing a compound PEO disinfectant is extremely necessary. In view of this, we systematically evaluated inhibition efficacy of a compound PEO disinfectant (designated as Lei-Huo-Fu) against bacteria, virus, and mycoplasma in the present study. The PEO disinfectant used in this study is primarily composed of Litsea cubeba, cinnamon, and star anise. These components have been widely studied. Litsea cubeba essential oil has demonstrated strong antioxidant, antibacterial, and insecticidal properties and exhibits potent antibacterial activity against various foodborne pathogens [23,24]. Cinnamon essential oil, when used as a feed additive, has shown beneficial effects on poultry performance, immunity, and antibacterial activity, making it a potential alternative to antibiotics [25]. Star anise oil has been found to effectively inhibit avian *Salmonella* in vitro, helping to prevent yellow-feathered chickens from *Salmonella* infection [26]. However, there have been no research reports on the disinfectant efficacy of PEOs blend extracted from these three components, and there are also few studies on the mycoplasma killing effect of PEOs.

Herein, this study aimed to systematically evaluate the in vitro inhibitory effect of the PEO disinfectant of Lei-Huo-Fu against *Escherichia coli*, *Staphylococcus aureus*, *Salmonella* spp., avian infectious bronchitis virus (IBV), as well as avian *Mycoplasma gallisepticum* (MG) and *Mycoplasma synoviae* (MS). Our results showed that the PEO disinfectant has remarkable inhibition efficacy against bacteria, virus, and mycoplasma, suggesting that it may have the potential to be developed as a novel broad-spectrum disinfectant, pending further in vivo and clinical validation.

## 2. Materials and Methods

### 2.1. Materials

The commercial compound plant essential oil (PEO) disinfectant, designated as Lei-Huo-Fu, was provided by Guangxi Huatan Technology Co., Ltd. (Nanning, China). Its precise formulation, as per the manufacturer, consists of 5% (*v*/*v*) cinnamon oil, 3% (*v*/*v*) Litsea cubeba oil, and 0.2% (*v*/*v*) peppermint oil. The formulation includes a mixture of glycerides, ethanol, and Tween 80 as solubilizers and emulsifiers, bringing the total active PEO content to 8.2% (*v*/*v*), the remainder is deionized water. Dey/Engley (D/E) neutralizing broth containing Tween 80 (Qingdao Hope Bio-Technology Co., Ltd., Shandong, China) and Frey’s broth medium (Beijing Zhonghai Biotech Co., Ltd., Beijing, China) were used. Tested microorganisms included standard strains and clinical isolates. The standard strains of *Escherichia coli (E. coli*) 8099, *Staphylococcus aureus* (*S. aureus*) ATCC 6538, infectious bronchitis virus (IBV) AV1511 M41 (Mass-type, GI-1), *Mycoplasma gallisepticum* (MG), and *Mycoplasma synoviae* (MS) were obtained from the China Institute of Veterinary Drug Control. The clinical isolates of *E. coli* (undefined pathogenicity type), *Salmonella* spp. (undefined pathogenicity type, serotype uncharacterized), and IBV GX-YL5 (LX4-type, GI-19) (accession number: HQ848267) and GX-NN200723 (Taiwan-type, GI-7) (accession number: MZ169543) were isolated and preserved by our laboratory [27,28].

### 2.2. Antibacterial Activity

#### 2.2.1. Neutralizing Agent Identification

The neutralizing efficacy of Tween 80 (in D/E broth) and its ability to rule out carryover antimicrobial effects were validated using a quantitative bactericidal test with six parallel groups (Table 1), as mandated by the ⟪Disinfection Technical Specification⟫ [29]. This experimental design confirms the following: (1) the neutralizer itself is not antimicrobial; (2) it effectively neutralizes the disinfectant’s activity; and (3) any residual inhibition is due to the disinfectant’s action and not the test system.

#### 2.2.2. Determination of Minimum Inhibitory Concentration (MIC)

The bacterial inoculum was prepared to a concentration of approximately 1 × 10^6^ CFU/mL, as verified by colony counting on agar plates. All assays included quality control strains (*E. coli* 8099 and *S. aureus* ATCC 6538) and were performed in triplicate on three separate occasions to ensure reproducibility. The methodology followed the guidelines of the ⟪Disinfection Technical Specification⟫ [29]. 4.5 mL of PEO disinfectant solutions of different concentrations (ranging from 0.06 µg/mL to 0.001875 µg/mL with continuously 2-fold dilution and dissolved in nutritional broth) were, respectively, mixed with 0.5 mL of *E. coli*, *S. aureus*, and *Salmonella* spp. cultures with a concentration of 1 × 10^6^ CFU/mL. The nutrient broth medium was used as the negative control and the bacterial culture as the positive control. After incubation at 37 °C for 24 h, the turbidity of the broth medium was observed. The MIC was defined as the lowest concentration inhibiting visible growth.

#### 2.2.3. Determination of Optimal Disinfection Concentration

Referring to the method of MIC, different concentrations of PEO disinfectant were mixed evenly with *E. coli*, *S. aureus*, and *Salmonella* spp. cultures, respectively, and then left to act at room temperature for 30 min. After that, D/E neutralizing broth was added to neutralize the disinfectant. The samples were then inoculated onto solid media and incubated at 37 °C for 24 h. The settings for negative and positive controls were the same as those in the MIC test. The bacterial counts in the plates before and after sterilization were recorded. The bactericidal rates (Ministry of Health Legal System and Supervision Department, 2002) were calculated to determine the effective inhibitory concentrations of PEO disinfectant against bacteria via the following formula: Bactericidal rate = [(Total bacterial count on plate before sterilization − Total bacterial count on plate after sterilization)/Total bacterial count on plate before sterilization] × 100%.

#### 2.2.4. Determination of Optimal Disinfection Time

Referring to the method of the optimal disinfection concentration, 0.015 μg/mL of PEO disinfectant solution was mixed evenly with three common bacteria, respectively, and then acted at room temperature for 10, 20, 30, 40, 50, and 60 min to explore the sterilization rates at different disinfection times.

### 2.3. Anti-IBV Activity

#### 2.3.1. Toxicity Testing for Embryonated Chicken Eggs

Ten-day-old SPF chicken embryos (5 embryos in each group) were inoculated via allantoic cavity route with 0.2 mL of diluted PEO disinfectant solution of different concentrations (ranging from 0.06 µg/mL to 0.001875 µg/mL with continuously 2-fold dilution and dissolved in PBS). PBS was used as the negative control. Embryo viability was determined after 120 h at 37 °C; The non-toxic concentration was selected for the subsequent antiviral test. The chicken embryo experiment was approved by the Animal Ethics Committee of Guangxi University (GXU-2023-308).

#### 2.3.2. Determination of Optimal Disinfection Concentration

After mixing different concentrations of PEO disinfectants that were non-toxic to chicken embryos (0.015, 0.0075, 0.00375 µg/mL) with equal volumes of IBV strains (M41, GX-YL5, GX-NN200723) for 60 min, 0.2 mL was taken and inoculated into SPF eggs via the allantoic cavity route (*n* = 5 per concentration/virus). The negative and positive controls were, respectively, inoculated with equal volumes of PBS or viral allantoic fluid. The allantoic fluids were collected after incubation at 37 °C for 120 h for the determination of viral loads by the RT-qPCR method established by our research group [30]. Briefly, viral RNA was extracted from the allantoic fluid using a commercial kit. Subsequently, the RNA was reverse transcribed into cDNA, which was then amplified by qPCR using specific primers targeting the nucleocapsid (N) gene of IBV. The viral titer was calculated based on a standard curve.

#### 2.3.3. Determination of Optimal Disinfection Time

Based on the toxicity results of the above PEO disinfectant, the maximum non-toxic concentration of 0.015 µg/mL PEO disinfectant was selected and treated with different strains of IBV of the same volume (M41, GX-YL5 and GX-NN200723) for different durations (10, 20, 30, 40, 50 and 60 min), respectively. Then, 120 h after inoculating the chicken embryos, the allantoic fluid was collected to detect the viral loads.

### 2.4. Anti-Mycoplasma Activity

#### 2.4.1. Resuscitation Culture of Mycoplasma and Determination of CCU

MG and MS strains were resuscitated in Frey’s broth (37 °C, 5–7 d) and subcultured when the color changed (from red to orange-yellow). Subsequently, MG and MS were continuously diluted 10 times and the color changing unit (CCU) was determined through the 96-well microtiter plate under microaerophilic humid conditions (10% CO_2_, 37 °C, 5–8 d). The highest dilution showing a change in color was defined as the CCU/mL for this strain.

#### 2.4.2. Determination of the MIC of Mycoplasma

Mycoplasma suspensions (adjusted to 1 × 10^4^ CCU/mL) were mixed with different concentrations of PEO disinfectant (ranging from 0.06 µg/mL to 0.001875 µg/mL with continuously 2-fold dilution and dissolved in Frey’s broth) in 96-well plates (0.1 mL/well). Controls included mycoplasma (Positive) and Frey’s broth (Negative). Plates were incubated microaerophilically (37 °C, 7–10 d). The MIC was the lowest disinfectant concentration preventing color change compared to the positive control.

### 2.5. Statistical Analysis

Data are expressed as mean ± standard deviation (SD). Differences were analyzed using one-way ANOVA followed by Dunnett’s multiple comparisons test (GraphPad Prism 9). Significance is denoted: ^##^ (*p* < 0.01, Positive vs. Negative control); ** (*p* < 0.01, Experimental vs. Positive control). Complete statistical details, including F-values, degrees of freedom, and precise *p* values for all comparisons, have been provided.

## 3. Results

### 3.1. Antibacterial Activity

#### 3.1.1. Neutralizing Agent Identification

The results (Table 1) showed that the Tween 80 did not exhibit any antimicrobial activity, and was effective in neutralizing the residual antimicrobial effects of the PEO disinfectant, and any reaction products formed between the PEO disinfectant and the neutralizing agent also lacked antimicrobial properties. Therefore, Tween 80 was a suitable neutralizer for the PEO disinfectant.

#### 3.1.2. Results of MIC Determination for Bacteria

The MIC values of PEO disinfectant for *E. coli*, *S. aureus*, and *Salmonella* spp. are shown in Table 2. The negative control medium remained transparent, indicating the absence of visible growth. The positive control medium was turbid and showed bacterial growth. PEO disinfectant exhibited varying degrees of growth inhibition against all tested strains, with MICs ranging from 0.00375 µg/mL to 0.03 µg/mL. Notable inter-strain variations were observed: MICs against standard strain *E. coli* (8099) and clinical isolate were 0.015 µg/mL and 0.00375 µg/mL, respectively, indicating that the PEO disinfectant has superior antimicrobial efficacy against the clinical isolate. The MICs of standard strain *S. aureus* (ATCC 6538) and *Salmonella* spp. isolate were 0.03 µg/mL and 0.00375 µg/mL, respectively.

#### 3.1.3. Optimal Disinfection Concentration for Bacteria

The bactericidal effects of PEO disinfectant at different concentrations were presented in Table 3. No bacterial growth was observed in the negative control medium, while bacterial growth was detected in the positive control medium. As the concentration of PEO disinfectant increased from 0.001875 µg/mL to 0.06 µg/mL, its bactericidal rate against *E. coli* standard strain (8099) increased from 61.26% to 99.30%, and its bactericidal rate against *E. coli* isolate increased from 77.23% to 99.63%. Notably, the PEO disinfectant showed the highest antibacterial activity against *S. aureus* (ATCC 6538), with bactericidal rate escalating from 61.26% to 99.83%. The PEO disinfectant also showed significant antibacterial activity against *Salmonella* spp. isolate, and the bactericidal rate increased from 69.22% to 98.47%.

#### 3.1.4. Optimal Disinfection Time for Bacteria

Based on results of the PEO disinfectant’s antibacterial activity, the PEO disinfectant with a concentration of 0.03 µg/mL was selected for the determination of optimal disinfection time. The results showed that no bacterial growth was observed in the negative control medium, while bacterial growth was detected in the positive control medium. With the extension of the disinfection time, the bactericidal rates gradually increased (Table 4). When the disinfection time reached more than 20 min, except that the bactericidal rate of PEO disinfectant against *Salmonella* spp. isolates was 85.97%, those of PEO disinfectant against the other three bacteria were all greater than 91%. When the disinfection time reached more than 30 min, the bactericidal rates against the four tested bacteria were all greater than 95%. When the reaction time increased from 10 min to 60 min, the antibacterial rates against the standard strain of *E. coli* (8099), *Escherichia coli* isolate, the standard strain of *S. aureus* (ATCC 6538), and *Salmonella* spp. isolate increased from 84.11% to 99.48%, from 88.04% to 99.22%, from 86.64% to 98.63%, and from 78.42% to 98.31%, respectively.

### 3.2. Anti-IBV Activity

#### 3.2.1. Toxicity Testing for Embryonated Chicken Eggs

The toxicity assessment results showed that when the concentration of the PEO disinfectant was ≥0.03 µg/mL, the chicken embryos exhibited varying degrees of lethality, stunting, and hemorrhaging (Table 5). However, when the concentrations of PEO disinfectant ranged from 0.00375 µg/mL to 0.015 µg/mL, the chicken embryos developed normally, indicating that the PEOs at concentrations within this range have no toxicity effects on chicken embryos. Therefore, the concentrations ranging from 0.00375 µg/mL to 0.015 µg/mL of PEO disinfectant were selected for subsequent observation of antiviral effects.

#### 3.2.2. Optimal Disinfection Concentration for IBV

**Figure 1:**
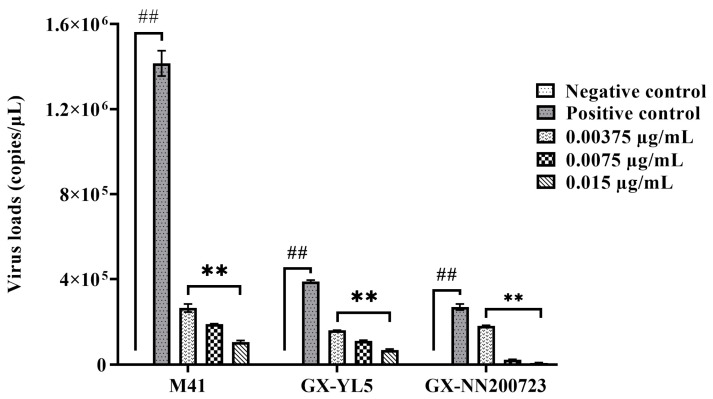
Virus loads after the action of different concentrations of PEO disinfectant and strains. Data are presented as mean ± SD (*n* = 5). Note: ^##^ indicates that there is statistical significance between the positive control group and the negative control group (*p* < 0.01). ** represents *p* < 0.01 between the experimental group and the positive control group.

According to the toxicity assessment results, the PEO disinfectants at concentrations of 0.015 µg/mL, 0.0075 µg/mL, and 0.00375 µg/mL were chosen for the observation of antiviral effects. The results showed that no virus was detected in the negative control by the highly sensitive RT-qPCR assay (limit of detection: 1 × 10^1^ copies/µL) [30], and PEO disinfectant treatment decreased viral loads in a dose-dependent manner. Compared to the positive control, PEO disinfectant at concentrations of 0.015 µg/mL, 0.0075 µg/mL, and 0.00375 µg/mL significantly reduced viral loads (*p* < 0.01) (Figure 1). For the strain M41 of IBV, the inactivation rates were significantly reduced by 99.9%, 86.7%, and 81.3%, respectively. For the strain GX-YL5, the inactivation rates were significantly reduced by 99.9%, 71.6%, and 58.8%, respectively. For the strain GX-NN200723, the inactivation rates were significantly reduced by 99.9%, 91.6%, and 32.9%, respectively.

#### 3.2.3. Optimal Disinfection Time for IBV

**Figure 2:**
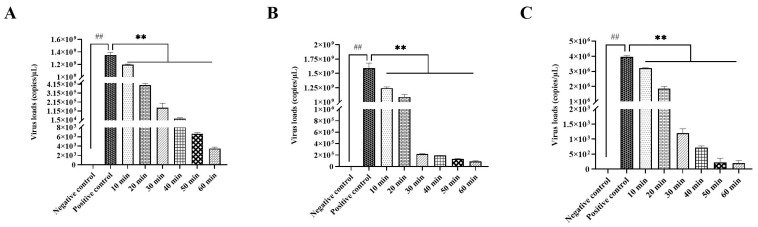
Virus loads of essential oils and equivalent viruses at different times of action. (**A**) With the same amount of IBV strain M41 fluid for different times. (**B**) With the same amount of IBV strain GX-YL5 fluid for different times. (**C**) With the same amount of IBV strain GX-NN200723 fluid for different times. Data are presented as mean ± SD (*n* = 5). Note: ^##^ indicates a significant difference (*p* < 0.01) between the positive control group and the negative control group. ** represents a significant difference (*p* < 0.01) between the experimental group and the positive control group.

Based on the above antiviral results of the PEO disinfectant, 0.015 µg/mL PEO disinfectant was selected to assess the optimal disinfection time. The results showed that no virus was detected in the negative control. Compared with the positive control, the 0.015 µg/mL PEO disinfectant significantly reduced the viral loads (*p* < 0.01) in a time-dependent manner. For the strain M41 of IBV, 0.015 µg/mL PEO disinfectant treatment for 10 min, 20 min, and 30 min could decrease viral loads by 11.38%, 99.97%, and 99.99%, respectively. No infectious virus was detectable after 40–60 min treatment with 0.015 µg/mL PEO disinfectant (Figure 2A). For the strain GX-YL5, 0.015 µg/mL PEO disinfectant treatment for 10 min, 20 min, and 30 min could decrease viral loads by 21.85%, 31.53% and 99.99%, respectively. Complete viral inhibition was achieved after 30–60 min treatment with 0.015 µg/mL PEO disinfectant (Figure 2B). For the strain GX-NN200723, 0.015 µg/mL PEO disinfectant treatment for 10 min, 20 min, and 30 min could decrease viral loads by 19.08%, 53.13% and 99.97%, respectively. No infectious virus was detectable after 40–60 min treatment with 0.015 µg/mL PEO disinfectant (Figure 2C).

### 3.3. Anti-Mycoplasma Activity

#### 3.3.1. Results of Color-Changing Units (CCU)

**Figure 3:**
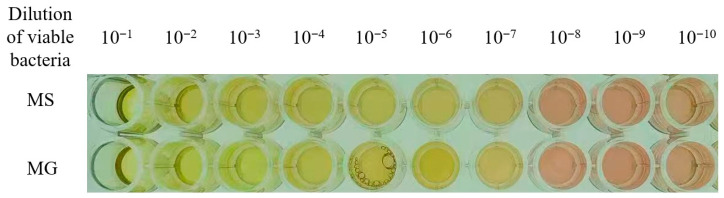
Results of color-changing units (CCU) determination by the mycoplasma culture was serially diluted 10-fold (from 10^−1^ to 10^−10^). A distinct color change in Frey’s broth medium from red to orange-yellow was defined as a positive result. No color change in the medium, which remained red, was defined as a negative result.

Resuscitation culture of mycoplasma showed that the Frey’s broth media has changed from red to a clear orange after 5 to 7 d of incubation, which was used as the visual threshold for a positive growth result. The resuscitated MG and MS were cultured to the logarithmic growth phase for the determination of CCU. The results of CCU determination were presented in Table 6. The broth media in wells with viable bacterial dilutions of 10^−1^ to 10^−7^ in the same row of the 96-well microtiter plate exhibited color change from red to clear orange, while the broth media in wells with dilutions of 10^−8^ to 10^−10^ showed no color change, indicating that the CCU of both MG and MS were determined to be 1 × 10^7^ CCU/mL (Figure 3).

#### 3.3.2. Results of MIC Determination for Mycoplasmas

**Figure 4:**
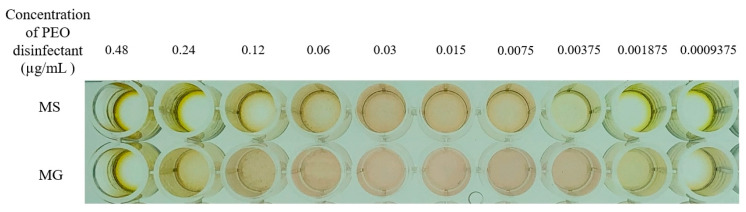
MIC determination results of PEO disinfectant for mycoplasma. A distinct color change in Frey’s broth medium from red to orange-yellow was defined as a positive result (indicating mycoplasma growth). No distinct color change in the medium, which remained red, was defined as a negative result (indicating growth inhibition). The yellow appearance in some wells at high disinfectant concentrations is attributed to the intrinsic color of the PEOs formulation.

The MIC values of PEO disinfectant against MG and MS are presented in Table 7. For MS, when the concentration of PEO disinfectant solution was 0.00375 µg/mL, the color of the corresponding broth medium changed from red to clear orange. For MG, when the concentration of PEO disinfectant solution was 0.001875 µg/mL, the color of the corresponding broth medium changed from red to clear orange (Figure 4). It is worth noting that as PEO disinfectant is a transparent yellow liquid, in the 96-well microtiter plate test, wells containing concentrations from 0.06 µg/mL to 0.48 µg/mL in the MS test and 0.12 µg/mL to 0.48 µg/mL in the MG test showed the yellow color of the disinfectant itself due to the high concentration of PEO disinfectant, which masked the red color of the culture medium (Figure 4). Therefore, the MIC for MG was 0.001875 µg/mL, and for MS, it was 0.00375 µg/mL.

## 4. Discussion

Animal infectious diseases (including zoonoses) have serious impacts on animal husbandry, food safety, and human health [31]. Disinfectants are widely used in the breeding industry, but traditional disinfectants generally have problems such as strong irritation, strong corrosiveness, easy development of drug resistance, and environmental pollution [32]. With the intensification of the drug resistance problem of traditional chemical disinfectants and the improvement of people’s environmental protection and health awareness, the development of natural, efficient, and low-toxicity alternative disinfection products has become a research hotspot [33]. PEOs have attracted much attention due to their broad-spectrum antimicrobial activity and low environmental toxicity [34]. In this study we investigated the inhibition efficacy of the PEO disinfectant (Lei-Huo-Fu) against common bacteria, virus, and mycoplasma. It was found that this PEO disinfectant has a broad spectrum and significant inhibitory activity against common pathogenic microorganisms and showed promising in vitro efficacy, suggesting its potential as a candidate for development into a natural disinfectant, though this requires further in vivo validation. Based on the known properties of its key constituents (e.g., cinnamaldehyde, citral) and previous literature on plant essential oils, its possible mechanisms of action include destroying the membrane structure of microorganisms [35], inhibiting energy metabolism [36], interfering with the synthesis of genetic material [37], etc. However, it is crucial to note that these specific mechanisms are hypothesized based on indirect evidence and require direct experimental validation in future work. This multi-target characteristic can effectively reduce the risk of drug resistance [38]. To the best of our knowledge, this is the first study to observe the inhibitory effects of essential oil disinfectants on bacteria, viruses, and mycoplasma.

Diseases caused by bacteria have brought serious economic losses to the breeding industry, and some of them are foodborne bacteria. Foodborne pathogens pose a substantial threat not only to the livestock industry but also to public health. Therefore, it is very necessary to choose safe and non-toxic disinfectants. PEOs has been demonstrated to have broad-spectrum antimicrobial effects against both Gram-positive and Gram-negative bacteria, including foodborne pathogens such as *Salmonella typhimurium* and *Listeria monocytogenes* [39]. Our findings indicated that the PEOs exhibit inhibitory effects against standard and isolated strains of *E. coli*, standard strain of *S. aureus*, and isolated strain of *Salmonella* spp., indicating that the PEO disinfectant can inhibit both Gram-positive and Gram-negative bacteria, which is consistent with the results of existing studies [39,40]. Notably, comparisons between standard and isolates of *E. coli* demonstrated the practical applicability of PEOs in real-world settings. In previous studies, the MIC value of litsea cubeba essential oil against *Escherichia coli* was 0.9 µg/mL [41]. The MIC value of cinnamon essential oil against *E. coli* and *S. aureus* was 1.0 mg/mL [42]. In this study, the MIC values of cubeba, cinnamon, anise, and other natural plant compound PEOs against *E. coli* and *S. aureus* were 0.015 µg/mL and 0.00375 µg/mL, respectively, reflecting the advantages of compound essential oils. In the future, this PEO disinfectant will be applied to more strains, including emerging zoonotic pathogens and multidrug-resistant strains, especially those that are prevalent in clinical practice.

In addition to the inhibitory effect of PEO disinfectant against bacteria, we also observed the inhibitory effect of this PEO disinfectant against viruses. Given the significant economic losses caused by the coronavirus IBV to the global poultry industry [43], we have chosen IBV to conduct experiments. The results indicated that the PEOs showed significant inhibitory activity against the three dominant epidemic strains of IBV M41 (Mass-type, GI-1), GX-YL5 (LX4-type, GI-19), and GX-NN200723 (Taiwan-type, GI-7), indicating its potential as an anti-IBV disinfectant. Notably, the reduction rate of viral load by PEO disinfectant exceeded the inactivation rate of 95.83% of the human coronavirus OC43 (HCoV-OC43) by Tea tree oil (TTO) at a concentration of 3.33% after 30 min of action [44]. While this PEO demonstrated a clear time-dependent effect, the assessment of very short contact times (e.g., 1–5 min), as recommended by guidelines for practical disinfection protocols, was not included. Investigating the rapid kinetics of Lei-Huo-Fu is a crucial next step for its application in scenarios requiring fast turnover, and this constitutes a key objective of our ongoing research. Previous studies have described various antiviral mechanisms of plant extracts against IBV. The virucidal activity of PEOs is attributed to its lipophilic properties, which disrupt the viral membrane or interfere with viral envelope proteins involved in host cell attachment [45]. However, this proposed mechanism requires direct experimental validation. These plant extracts are capable of penetrating the viral membrane and disrupting its structural integrity, leading to membrane rupture and subsequent viral inactivation. PEOs and their phytochemical components interfere with viral replication and also benefit the host’s respiratory system through mucolysis and bronchiectasis [46]. Another study by our research group found that PEOs have significant anti-IBV properties, reducing morbidity, inhibiting the IBV mRNA expression level, and improving cure rates [47]. However, the specific antiviral mechanisms still need to be further explored. In the future, it will be necessary to apply this disinfectant to more viruses, such as avian influenza virus or porcine epidemic diarrhea virus. Viruses include enveloped viruses and unenveloped viruses [48]. In the future, this disinfectant can also be applied the unenveloped viruses.

Mycoplasma disease is currently a worldwide problem. Especially in recent years, mycoplasma infections in China have been very serious [49,50]. In 2023, mycoplasma disease was added to the national list of clean diseases in China. Mycoplasma, as the smallest prokaryotic microorganism without a cell wall, has natural resistance to most disinfectants targeting the cell wall [51]. At present, research on the inhibitory effect of PEOs on mycoplasma is particularly rare. Therefore, it is extremely urgent to develop disinfectants suitable for mycoplasma. The previous study showed that clove, cumin, and cinnamon oils inhibited the growth of clinical isolates of MG and MS with MIC values ranging from 0.49 to 15.63 µg/mL [52]. In our study, the MIC of PEOs for MG and MS ranged from 0.001875 µg/mL to 0.00375 µg/mL. Therefore, it is feasible to use PEOs as mycoplasma disinfectants. This study only observed the inhibitory effect of PEO disinfectant on MG and MS in poultry. It is necessary to investigate its inhibitory effect on mycoplasmas from different animals in the future. In addition, the mechanism by which PEOs inhibits mycoplasma remains to be further studied.

In summary, our study indicates that the PEO disinfectant (Lei-Huo-Fu) has a significant inhibitory effect on bacteria, viruses, and mycoplasma, providing a theoretical and practical basis for the development of natural disinfectants. The PEO disinfectant (Lei-Huo-Fu) is particularly applicable to the prevention and treatment of livestock and poultry diseases under China’s ban on antibiotics policy. Of course, this study has several limitations that should be acknowledged. Firstly, the exact mechanistic pathways of Lei-Huo-Fu were not empirically verified; the proposed mechanisms (e.g., membrane disruption) remain hypotheses based on the literature and require validation through future experiments such as electron microscopy (TEM/SEM), cellular leakage assays, and/or viral genome quantification assays. Secondly, this work is confined to in vitro assessments under controlled laboratory conditions. Specifically, the antiviral assays were conducted exclusively in embryonated chicken eggs, a standard model for IBV propagation but one that lacks the complexity of a live host. Formal in vivo safety and efficacy trials, as well as evaluation against fungal pathogens and non-enveloped viruses, are necessary to fully establish its utility. Finally, critical parameters for practical deployment, including shelf-life stability, efficacy in the presence of organic load, and compatibility with common materials, were not investigated here.

Therefore, the immediate priorities for future work will be to do as follow: (1) elucidate the antimicrobial mechanism using the aforementioned biochemical and microscopic techniques; (2) conduct in vivo trials in relevant animal models to confirm this efficacy in the clinically relevant context; (3) expand the pathogen panel to include fungi and non-enveloped viruses; and (4) comprehensively evaluate the product’s stability and performance under realistic environmental conditions. Despite these limitations, our findings robustly demonstrate the potent in vitro, broad-spectrum efficacy of Lei-Huo-Fu against a notable range of pathogens, providing a strong justification for these further investigations. The current study thus provides compelling in vitro evidence for the efficacy of Lei-Huo-Fu, while clearly framing its limitations to guide future research.

## 5. Conclusions

In conclusion, the compound PEO disinfectant (Lei-Huo-Fu) demonstrates broad-spectrum efficacy against the tested panel of bacteria (including *E. coli*, *S. aureus*, and *Salmonella* spp.), viruses (IBV), and mycoplasmas (MG, MS), and shows significant potential for future development as a broad-spectrum disinfectant, subject to successful outcomes from necessary in vivo and field studies. It is highly necessary to conduct in-depth research on the mechanism of action, in vivo experiments, and safety evaluations, as well as research into the long-term stability of PEO disinfectants, in the future.

## Figures and Tables

**Table 1:** Identification result of neutralizing agent of PEO disinfectant.

Experimental Groups	Results
Broth	Clear
Broth + bacterial suspension	Turbid
Disinfectant + Bacterial suspension	Clear
Neutralizing agent + Bacterial suspension	Turbid
(Disinfectant + Bacterial suspension) + Neutralizing agent	Clear
(Disinfectant + Neutralizing agent) + Bacterial suspension	Turbid

**Table 2:** MIC determination results of PEO disinfectant for bacteria.

Bacteria Strains	Negative Control	Positive Control 0.000000 µg/mL	Concentrations of PEO Disinfectant					
			0.001875 µg/mL	0.00375 µg/mL	0.0075 µg/mL	0.015 µg/mL	0.03 µg/mL	0.06 µg/mL
*Escherichia coli* (8099)	−	+	+	+	+	−	−	−
*Escherichia coli* (isolate)	−	+	+	−	−	−	−	−
*Staphylococcus aureus* (ATCC 6538)	−	+	+	−	−	−	−	−
*Salmonella* spp. (isolate)	−	+	+	+	+	+	−	−

Note: “+” indicates visible turbidity (positive growth), and “−” indicates a clear solution (no visible growth).

**Table 3:** Bactericidal rates of PEO disinfectant at different concentrations.

Bacterial Strains	Negative Control	Positive Control 0.000000 µg/mL	Bactericidal Rates of PEO Disinfectant at Different Concentrations						df (Between Groups/ Within Groups)	F	*p*
			0.001875 µg/mL	0.00375 µg/mL	0.0075 µg/mL	0.015 µg/mL	0.03 µg/mL	0.06 µg/mL			
*Escherichia coli* (8099)	−	00.00%	61.26%	85.86%	94.59%	97.38%	98.78%	99.30%	5/48	569.1	<0.001
*Escherichia coli* (isolate)	−	00.00%	77.23%	89.30%	94.97%	98.83%	99.20%	99.63%	5/48	621.5	<0.001
*Staphylococcus aureus* (ATCC 6538)	−	00.00%	61.26%	95.03%	95.72%	96.58%	98.63%	99.83%	5/48	712.3	<0.001
*Salmonella* spp. (isolate)	−	00.00%	69.22%	78.97%	88.97%	89.74%	97.86%	98.47%	5/48	598.2	<0.001

Note: Data are means of triplicate measurements from three independent experiments (*n* = 9 per concentration/time point). “−” indicates a clear solution (no visible growth).

**Table 4:** The determination results of the optimal disinfection time of PEO disinfectant against bacteria.

Bacterial Strains	Negative Control	Positive Control 0 min	Bactericidal Rates at Different Exposure Times						df (Between Groups/ Within Groups)	F	*p*
			10 min	20 min	30 min	40 min	50 min	60 min			
*Escherichia coli* (8099)	−	00.00%	84.11%	91.56%	97.55%	97.90%	98.78%	99.48%	5/48	426.4	<0.001
*Escherichia coli* (isolates)	−	00.00%	88.04%	91.72%	97.04%	97.30%	98.75%	99.22%	5/48	458.7	<0.001
*Staphylococcus aureus* (ATCC 6538)	−	00.00%	86.64%	91.72%	95.21%	96.40%	97.09%	98.63%	5/48	492.1	<0.001
*Salmonella* spp. (isolate)	−	00.00%	78.42%	85.97%	96.25%	97.16%	97.94%	98.31%	5/48	475.3	<0.001

Note: Data are means of triplicate measurements from three independent experiments (*n* = 9 per concentration/time point). “−” indicates a clear solution (no visible growth).

**Table 5:** Results of the effects of different concentrations of PEO disinfectant on chicken embryos.

Parallelism Degree	Different Concentrations of PEOs Disinfectant				
	0.00375 µg/mL	0.0075 µg/mL	0.015 µg/mL	0.03 µg/mL	0.06 µg/mL
1	−	−	−	+	+
2	−	−	−	−	+
3	−	−	−	+	+
4	−	−	−	+	+
5	−	−	−	+	+

Note: “+” indicates chicken embryos exhibiting lethality, stunting, or hemorrhaging, and “−” indicates unaffected chicken embryos.

**Table 6:** Results of color-changing units (CCU) determination.

Mycoplasma Strains	Negative Control	Dilution of Viable Bacteria									
		10^−1^	10^−2^	10^−3^	10^−4^	10^−5^	10^−6^	10^−7^	10^−8^	10^−9^	10^−10^
MG	−	+	+	+	+	+	+	+	−	−	−
MS	−	+	+	+	+	+	+	+	−	−	−

Note: “+” indicates a distinct color change in the medium from red to orange-yellow (positive growth), and “−” indicates no color change (no growth).

**Table 7:** MIC determination results of PEO disinfectant for mycoplasmas.

Mycoplas-ma Strains	Negative Control	Positive Control	Concentrations of PEOs Disinfectant									
		0.000000 µg /mL	0.48 µg /mL	0.24 µg /mL	0.12 µg /mL	0.06 µg /mL	0.03 µg /mL	0.015 µg /mL	0.0075 µg /mL	0.00375 µg /mL	0.001875 µg /mL	0.000938 µg /mL
MG	−	+	−	−	−	−	−	−	−	−	+	+
MS	−	+	−	−	−	−	−	−	−	+	+	+

Note: “+” indicates a distinct color change in the medium from red to orange-yellow (positive growth), and “−” indicates no color change (no growth).

## Data Availability

The original contributions presented in this study are included in the article. Further inquiries can be directed to the corresponding author.
